# Sedentary Behavior and Alcohol Consumption Increase Breast Cancer Risk Regardless of Menopausal Status: A Case-Control Study

**DOI:** 10.3390/nu11081871

**Published:** 2019-08-12

**Authors:** Jordana Carolina Marques Godinho-Mota, Larissa Vaz Gonçalves, Joao Felipe Mota, Leonardo Ribeiro Soares, Raquel Machado Schincaglia, Karine Anusca Martins, Ruffo Freitas-Junior

**Affiliations:** 1 Centro Avançado de Diagnóstico da Mama, Hospital das Clínicas, Federal University of Goiás, Goiania-GO 74.605-020, Brazil; 2 Faculty of Nutrition, Federal University of Goiás, St. 227, Block 68, Goiania-GO 74.605-080, Brazil; 3 Clinical and Sports Nutrition Research Laboratory (Labince), Faculty of Nutrition, Federal University of Goiás, St. 227, Block 68, Goiania-GO 74.605-080, Brazil

**Keywords:** breast neoplasm, risk factors, cancer prevention, lifestyle, premenopausal, postmenopausal

## Abstract

Identification of modifiable risk factors for breast cancer is critical for primary prevention of the disease. The aim of this study was to evaluate how certain lifestyle variables modify the chances of developing breast cancer based on menopausal status. A case-control study was performed in a group of 542 women, 197 who were diagnosed with breast cancer and 344 control individuals. The groups were matched by age, body mass index, and menopausal status. Participants were evaluated for level of physical activity, alcohol consumption, smoking habit, weight, height, and waist circumference (WC). A multivariate logistic regression model was used to estimate odds ratios and 95% confidence intervals (95% CI). Regular consumption of alcoholic beverages (2.91, 95% CI 1.58–5.38 and 1.86, 95% CI 1.15–3.03) and sedentary behavior (2.08; 95% CI 1.12–3.85 and 1.81; 95% CI 1.12–2.94) were associated with breast cancer risk in pre- and postmenopausal women, respectively. High WC (3.31, 95% CI 1.45–7.55) was associated with an increased risk of developing breast cancer in premenopausal women. While in postmenopausal women, current smoking (2.43, 95% CI 1.01–5.83) or previous history of smoking (1.90; 95% CI 1.14–3.14) increased the chances of developing breast cancer. Sedentary behavior and current consumption of alcoholic beverages were more likely to increase the risk of developing breast cancer regardless of menopausal status.

## 1. Introduction

Breast cancer represents a public health problem on a global scale [1]. Approximately 2 million new breast cancer cases and 626,000 deaths from the disease worldwide were estimated for the year 2018 by The International Agency for Research on Cancer (IARC) [1]. In Brazil, it is estimated that a total of 59,700 new cases will be diagnosed in 2019, corresponding to 29.5% of cancers in women, excluding non-melanoma skin cancer [2]. Regarding mortality, in 2016 there were 16,069 deaths from breast cancer in Brazil, with higher rates in the south and southeast regions [2].

Despite the high incidence and prevalence of this cancer, the overall survival of Brazilian women is increasing. Five-year survival estimates were 76.9% and 75.2% from 2005 to 2009 and 2010 to 2014, respectively [3]. The latest multicenter study showed a five-year survival rate of 88.74% of patients with a difference in staging, molecular subtypes, and immunohistochemistry [4].

Identification of the etiological factors for breast cancer is important for the primary prevention of the disease [5,6]. Only 10% of breast cancer cases are thought to be hereditary [7]. There is evidence for a strong association between lifestyle variables and increased breast cancer risk related to menopausal status [6,8,9]. Lifestyle variables most associated with breast cancer include physical inactivity, alcohol consumption, smoking, abdominal, and total adiposity [8,9,10,11,12,13,14,15,16]. However, data currently available in the literature remain inconclusive and are limited by differing sample populations and methodological variations.

In this context, identification of modifiable risk factors may help to elaborate specific strategies for the control and management of breast cancer. The aim of this study was to evaluate the effects of certain lifestyle variables on the chances of developing breast cancer based on menopausal status.

## 2. Materials and Methods

### 2.1. Design and Sample Size

This case-control study was conducted from August 2014 to January 2018. In total, 542 women were enrolled in the study. There were 197 participants diagnosed with breast cancer and 344 control individuals. Participants were matched by age, body mass index, and menopausal status. A post hoc sample size analysis was performed using G*Power 3.1.9.2 software (Heinrich Heine Universität, Düsseldorf, Germany). An odds ratio of 1.81 for the variable postmenopausal physical activity was obtained. The proportion of the outcome for the unexposed obtained was 55.56, type I error 0.05, returning a test power (1−β) of 99.9%, and demonstrating that a suitable sample for the analysis was determined. The study was approved by the Ethics Committee of the Federal University of Goias (protocol number 751.387/2014). All study participants received oral and written information about the study and gave their written informed consent to participate in the study.

### 2.2. Selection of Cases and Controls

Women aged between 30 and 80 years with new primary breast cancer diagnoses were recruited as cases for the study. Breast cancer originating in the breast parenchyma and that had not spread beyond the breast or the lymph nodes under the arm was considered as a primary breast cancer. The participants were evaluated immediately after histological diagnoses and before systemic treatment. Women diagnosed with metastases, cutaneous neoplasms (melanoma and squamous cell carcinoma), lymphomas, ductal carcinoma in situ or with history of other cancers were not eligible for this study.

Healthy participants with similar values for body mass index (BMI), age, and menopausal stage to those in the case group were recruited as control individuals. The criteria used to determine eligibility for the control group were mammography and breasts clinical examination without pathological changes up to one year before recruitment, and absence of a personal history of breast cancer or other malignant neoplasia. The exclusion criteria for both groups were pregnancy, lactation, cognitive impairment, psychiatric illness, limb amputation or loss of mobility that would make it impossible to collect the necessary information.

### 2.3. Measurements

A personal interview was conducted by a trained researcher using a structured and standardized questionnaire. Age (full years), first-degree family history of breast cancer, marital status (with or without partner), and level of education attained (grade school, high school—complete/incomplete, and undergraduate degree) were obtained using the questionnaire.

Intensity of physical activity was measured using the short version of the International Questionnaire of Physical Activity (IPAQ) [16]. Women who reported moderate/vigorous intensity activities achieving a minimum of at least 600 MET-min/week were considered as active, and those who reported less than 600 MET-min/week) were considered as sedentary [16].

Smoking status was determined based on the response to the question, “Do you now or have you ever smoked cigarettes, at least one a day for one year’s time?” If the answer was “yes,” the participant reported the average number of cigarettes smoked per day [17]. Consumption of alcoholic drinks was calculated in grams per day according to frequency, quantity, and type of drink mentioned as habitual. Total alcohol intake was considered assuming 12.8 g of ethanol for 360 mL of regular beer, 11.3 g for 360 mL of light beer, 11.0 g for 120 mL of wine, and 14.0 g for 45 mL of liquor [18].

To ensure data reliability, measurement techniques recommended by Habicht [19] were applied in anthropometric evaluations. A stadiometer accurate to 0.1 cm was used to measure height. Participants were required to wear light clothing for body weight measurements. A digital scale accurate to 0.1 kg and with a capacity of 150 kg was used to evaluate body weight. BMI was calculated using the formula: Weight (kg)/height (m)^2^ [20]. WC was measured on undressed participants with an inelastic tape placed at the midpoint between the anterior superior iliac crest and the last rib [21], and classified as normal (<80 cm) or elevated (≥80 cm) [22].

### 2.4. Quality Control

All interviewers and kinanthropometrists were previously trained and standardized in a pilot study to ensure data quality. In addition, study data were checked on three occasions; by the interviewer, by the study coordinator, and at the moment of database entry.

### 2.5. Statistical Analysis

Data were processed under the double entry method using Excel software 10.0 (version 2013, Microsoft Corporation, Redmond, WA, USA) and consistency was verified by Epi-Info™ 2014 software (version 7.1.5, Centers for Disease Control and Prevention, Atlanta, GA, USA). Statistical analysis was performed using STATA^®^ software (version 14.0, StataCorp, College Station, TX, USA). The Shapiro–Wilk test was used to test normality and the Mann–Whitney test was used to compare groups. Data were reported as mean ± standard deviation.

Categorical variables were expressed in absolute and relative frequencies. The Pearson’s chi square test or Fisher’s exact two-tailed test were used to assess the degree of homogeneity or comparability between groups. Odds ratios and 95% confidence interval (95% CI) for developing breast cancer based on menopausal status were calculated. Binary logistic regression and multiple logistic regression calculations were performed using the backward method (*p* < 0.20) and adjusted by BMI according to the automatic selection of the statistical program. The data were expressed in the form of a Forest Plot. The level of significance used for all tests was 5%.

## 3. Results

Demographic characteristics are shown in Table 1. In postmenopausal women, the control group had a higher percentage of women with a completed undergraduate degree than the case group. No differences were observed based on age and marital status between both groups. Family history of breast cancer did not differ in pre- (cases: 10.0% vs. controls: 12.8%; *p* = 0.541) and postmenopausal stages (cases: 10.3% vs. controls: 10.4%; *p* = 0.930).

Considering the variables associated with lifestyle, women in the case group were mostly sedentary and reported higher consumption of cigarettes and alcoholic beverages, regardless of menopausal status (Table 2). In premenopausal status, the control group had an average higher body weight compared to case group (*p* = 0.03, Table 3).

**Figure 1:**
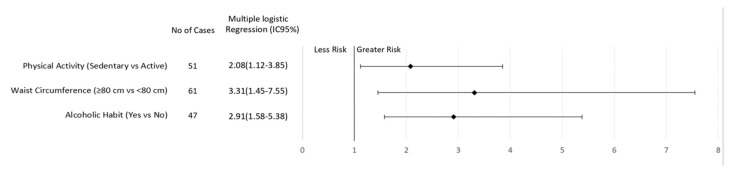
Association of lifestyle behavior and breast cancer risk in women according to premenopausal status.

**Figure 2:**
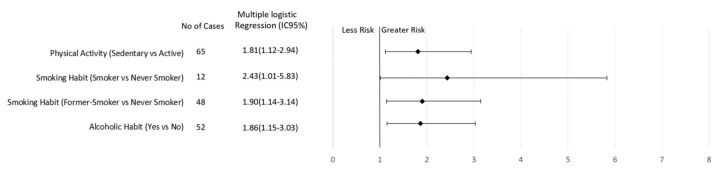
Association of lifestyle behavior and breast cancer risk in women according to postmenopausal status.

After multiple logical regression analyses, sedentary behavior and regular consumption of alcoholic beverages were found to be risk factors for breast cancer regardless of menopausal status (Figure 1 and Figure 2). In addition, elevated WC increased the risk of breast cancer in premenopausal women (3.31; CI 1.45–7.55, *p* = 0.005; Figure 1). On the other hand, current smoking (2.43, CI 1.01–5.83, *p* = 0.03) or a previous history of smoking (1.90, CI 1.14–3.14, *p* = 0.006) increased the risk of breast cancer in postmenopausal women (Figure 2).

## 4. Discussion

Our results provide a more comprehensive view of the relationship between lifestyle variables and breast cancer when menopausal status is considered. A sedentary lifestyle and regular consumption of alcoholic beverages both increased the risk of developing breast cancer, regardless of menopausal status. In addition, abdominal adiposity was associated with a 3.3-fold increase in the risk of developing breast cancer in premenopausal women. For postmenopausal women, a current or past history of smoking increased the risk of developing breast cancer.

Epidemiological studies have reported a consistent association between the regular consumption of alcoholic beverages and the development of breast cancer [23,24]. Following the re-analysis of individual data from 53 epidemiological studies which associated breast neoplasia with daily consumption of 10 g of ethanol [25], a meta-analysis found sufficient evidence that even light alcohol consumption can significantly increase disease risk [26]. Possible mechanisms for this link include high concentrations of bioactive free estrogen and androgens, a pro-inflammatory environment, and presence of plasma insulin-like growth factors which promote tumor progression [6,26].

Our study reinforces that lifestyle variables have different associations with breast cancer risk based on menopausal status [6,8,11,13,15,27,28]. To our knowledge, this is the first study to carry out an investigation into the effects of lifestyle variables on breast cancer risk based on the menopausal status in Brazilian women. This will allow for greater nationwide awareness about effects of these relationships on the health status of women. Prospective and population-based studies have demonstrated a direct relationship between a sedentary lifestyle and the risk of developing breast cancer [23,27,29,30]. However, this correlation may vary with age, menopausal status, and tumor biology [23,27,28,29,30,31,32,33,34,35]. In this study, the association was strongly inversed for both menopausal status and age.

The biological mechanisms by which physical activity may reduce the risk of breast cancer development remain uncertain [23,27,29,30]. However, these mechanisms are probably associated with maintenance or reduction of body weight, abdominal adiposity, serum estrogen levels, and insulin resistance [23,32,33,34]. In addition, sedentary behavior combined with other unhealthy habits such as smoking, high consumption of alcohol and saturated fatty acids may increase the risk for breast neoplasia [10,23,36].

In this study, WC was observed as a predictor for developing breast cancer in premenopausal women, regardless of BMI. Our findings confirm that the relationship between obesity and breast cancer is complex and shows divergence between ethnic groups [37]. After adjusting for BMI, WC became the most important adiposity marker in premenopausal women with breast cancer [38,39,40]. This may be explained by the relationship between central adiposity and high concentrations of cytokines, insulin, insulin-like growth factor 1 (IGF-1) [37], estradiol, and sex hormone binding globulin in premenopausal women [41].

Although smoking is considered an important risk factor for various types of cancer according to the International Agency for Research, its effects on breast cancer risk remain inconclusive [42]. However, recent studies found a positive association between smoking habits and breast cancer risk, which is consistent with our study [43,44]. Tobacco contains different chemical compounds which may contribute to carcinogenesis through various pathways, including induction of mutations, increased inflammation and oxidative stress, and epigenetic mechanisms [45]. It is suggested that over time these pro-inflammatory effects supersede those of anti-estrogens that mitigate breast cancer risk [46].

In a study conducted on a cohort of women from 17 countries in North and Latin America, Europe, and Asia, authors observed a 1.33-fold increase in the risk of developing breast cancer in smokers compared to non-smokers, without accounting for differences in the type of tumor and age of diagnosis [47]. Recent findings also indicate that the positive correlation increases with a greater number of cigarettes consumed per day and higher exposure time [47,48]. We found that smoking was associated with breast cancer only in the postmenopausal period, which may be explained by longer duration of exposure.

Some potential limitations of our study were the case-control study design, the assessment of physical activity level using a questionnaire, and the absence of food consumption analysis. In addition, Jewish ancestry, breast density, and radiation exposure from computed tomography were not evaluated, which could influence the results. Several studies have reported higher rates of breast cancer 1 and 2 (BRCA1 and 2) gene mutations in Ashkenazi Jewish women [49,50]. However, Ashkenazi women living in Brazilian cities showed a relatively lower than expected breast cancer mortality pattern [51]. The authors raised the hypothesis that germ mutations may be modulated by some environmental factors that perhaps could act as protective factors, delaying the mutation expression [51]. On the other hand, high breast density is considered a risk factor for breast cancer and have a synergistic interaction with overweight [52].

Our study concluded that a sedentary lifestyle and regular consumption of alcoholic beverages were considered risk factors for breast cancer in women, regardless of menopausal status. In addition, increased abdominal adiposity and smoking posed a risk for premenopausal and premenopausal breast cancer, respectively.

## Figures and Tables

**Table 1:** Demographic and economic characteristics among participants according to menopausal status.

	Premenopausal 213 (39.37)			Postmenopausal 328 (60.63)		
	Cases	Controls	*p*-Value	Cases	Controls	*p*-Value
	80 (37.56)	133 (62.44)		117 (35.67)	211 (64.33)	
Age (years)	41.84 ± 6.62	41.41 ± 7.01	0.488	59.21 ± 8.78	58.77 ± 8.22	0.722
Marital status			0.594			0.184
With partner	54 (67.50)	85 (63.91)		61 (52.14)	126 (59.72)	
Without partner	26 (32.50)	48 (36.09)		56 (47.86)	85 (40.28)	
Schooling			0.328 *			<0.001 *
Grade school	5 (6.33)	3 (2.34)		14 (12.73)	10 (5.03)	
Incomplete high school	9 (11.39)	13 (10.16)		44 (40.00)	49 (24.62)	
High school and undergrad course	65 (82.28)	112 (87.50)		52 (47.27)	140 (70.35)	

Values presented in absolute (*n*) and relative (%) frequencies or mean ± standard deviation. *p*-value–Pearson’s Chi-square test (* Fisher’s exact two-tailed test or Mann–Whitney test). Significance level of 5%.

**Table 2:** Lifestyle characteristics among participants according to menopausal status.

	Premenopausal 213 (39.37)			Postmenopausal 328 (60.63)		
	Cases	Controls	*p*-Value	Cases	Controls	*p*-Value
	80 (37.56)	133 (62.44)		117 (35.67)	211 (64.33)	
Physical activity level *			0.011			0.021
Active	29 (36.25)	73 (54.89)		52 (44.44)	123 (58.29)	
Sedentary	51 (63.75)	60 (45.11)		65 (55.56)	88 (41.71)	
Cigarette consumption (units/day)	16.18 ± 9.99	8.72 ± 4.90	<0.005	17.59 ± 12.48	9.22 ± 6.31	<0.001
Smoking habit *			0.271			0.005
Never smoker	58 (72.50)	108 (81.20)		57 (48.72)	141 (66.82)	
Smoker	6 (7.50)	5 (3.76)		12 (10.26)	12 (5.69)	
Ex-smoker	16 (20.00)	20 (15.04)		48 (41.03)	58 (27.49)	
Ethanol consumption (g/day)	1.59 ± 1.04	0.82 ± 0.35	<0.001	1.44 ± 0.76	0.90 ± 0.39	<0.001
Alcoholic habit *			0.001			0.016
Not	33 (41.25)	86 (64.66)		65 (55.56)	146 (69.19)	
Yes	47 (58.75)	47 (35.34)		52 (44.44)	65 (30.81)	

Values presented in absolute (*n*) and relative (%) or mean ± standard deviation frequencies. *p*-value–Pearson’s chi-square test (* Fisher’s exact two-tailed test or Mann–Whitney test). Significance level of 5%.

**Table 3:** Anthropometric characteristics among participants according to menopausal status.

	Premenopausal 213 (39.37)			Postmenopausal 328 (60.63)		
	Cases	Controls	*p*-Value	Cases	Controls	*p*-Value
	80 (37.56)	133 (62.44)		117 (35.67)	211 (64.33)	
Height (meters)	1.58 ± 0.06	1.60 ± 0.06	0.19	1.55 ± 0.06	1.57 ± 0.06	0.07
Body weight (kg)	66.01 ± 12.85	69.66 ± 13.84	0.04	68.37 ± 13.61	68.51 ± 13.14	0.78
BMI	26.26 ± 4.73	27.21 ± 5.34	0.19	28.28 ± 5.62	28.02 ± 5.09	0.96
Normal weight *	0	1 (0.75)	0.06	2 (1.71)	3 (1.42)	0.74
Overweight	40 (50.00)	85 (63.91)		79 (67.52)	151 (71.56)	
Obesity	40 (50.00)	47 (35.34)		36 (30.77)	57 (27.01)	
Waist circumference	87.61 ± 11.79	87.75 ± 12.79	0.97	93.33 ± 12.08	91.73 ± 12.66	0.39
Low risk (<80 cm) *	16 (20.78)	41 (32.54)	0.07	12 (10.91)	31 (15.05)	0.31
Increased risk (≥80 cm)	61 (79.22)	85 (67.46)		98 (89.09)	175 (84.95)	

Values presented in absolute (*n*) and relative (%) or mean ± standard deviation frequencies. *p*-value–(* Fisher’s exact two-tailed test; or Mann–Whitney test). Significance level of 5%.
